# The T350G Variation of Human Papillomavirus 16 *E6* Gene Prevails in Oropharyngeal Cancer from a Small Cohort of Greek Patients

**DOI:** 10.3390/v14081724

**Published:** 2022-08-04

**Authors:** Christine Kottaridi, Panagiota Resta, Danai Leventakou, Katerina Gioti, Ioannis Zygouras, Alina-Roxani Gouloumi, Georgios Sakagiannis, Khalid J. Alzahrani, Maria S. Venetikou, Fragkiski Anthouli-Anagnostopoulou, Apostolos Beloukas

**Affiliations:** 1 Department of Genetics, Development and Molecular Biology, School of Biology, Aristotle University of Thessaloniki, 54124 Thessaloniki, Greece; 2 Department of Biomedical Sciences, University of West Attica, 122 43 Athens, Greece; 3 National AIDS Reference Centre of Southern Greece, Department of Public Health Policy, University of West Attica, 115 21 Athens, Greece; 4 2nd Department of Pathology, University Hospital Attikon, School of Medicine, National and Kapodistrian University of Athens, 124 62 Athens, Greece; 5 ENT Consultant, 401 General Army Hospital, 115 25 Athens, Greece; 6 Department of Clinical Laboratories Sciences, College of Applied Medical Sciences, Taif University, P.O. Box 11099, Taif 21944, Saudi Arabia

**Keywords:** Human Papillomavirus, HPV, type 16, genetic variation, head and neck cancer

## Abstract

Recent trends have shown a dramatic rise in the incidence of oropharyngeal squamous cell carcinoma strongly associated with high-risk human papillomavirus (HPV) of type 16. The genetic variability of HPV16 has been extensively studied in cervical cancer but there are very limited published data concerning the genetic variations of this HPV type in oropharyngeal cancer. In the present study, the genetic variations of HPV16 *E6* gene sequences originated from a small cohort of Greek patients diagnosed with oropharyngeal cancer were assessed. The vast majority of the sequences clustered within the European variant branch. The T350G variation was found to be the predominant one. This finding may indicate the need for further studies that could explain the possible impact of this variant in the pathomechanisms of oropharyngeal cancer.

## 1. Introduction

Human Papilloma Virus (HPV) is a member of the Papillomaviridae (PV) family, and constitutes one of the earliest known viral families [1] that is capable of infecting the mucosal epithelium [2]. It is a small circular double-stranded DNA virus, with a genome of roughly 8000 bp, encapsidated in an icosahedral capsid [3]. The viral genome is divided into three domains: the early (E) region (genes *E1*, *E2*, *E4*, *E5*, *E6* and *E7*) encoding viral proteins responsible for the replication; the late (L) region, encoding the major (L1) and minor (L2) capsid proteins; and the non-coding region (NCR), or long control region (LCR), between the *L1* and *E6* genes [3].

According to The Papillomavirus Episteme (PaVE) [4], there are more than 400 and 200 reference genomes for Human and Animal papillomavirus, respectively, based on sequence records from the NCBI database. Around 40 different types can infect the genitalia and the anus and they are classified as low-risk [5] and high-risk (HR) based on their ability for malignant transformation [6]. High-risk types are connected with premalignant lesion development and certain types of cancer because of their high oncogenic activity. The most important high-risk types are the HPV-16, 18, 31, 33, 35, 39, 45, 51, 52, 56, 58 and 59 and they can cause differential manifestations to cancer of the cervix, head and neck, anus, vagina, vulva, and penis [7]. HPV is estimated to cause about 610,000 cancer cases and 250,000 deaths per year, respectively [8].

Head and neck cancer (HNC) includes a wide group of tumors that originated from the head and neck region, such us cancers from the oral cavity, nasopharynx, oropharynx, larynx, and hypopharynx [9]. The head and neck squamous cell carcinoma (HNSCC), is the most common type of HNC and the sixth most common cancer worldwide [10], with around 900,000 cases and 450,000 deaths in 2018, respectively [11]. It appears more frequently on older patients and it is generally associated with extensive use of tobacco and alcohol, but not in all cases. However, there is an increasing trend of detecting HPV genome among young patients with oropharyngeal tumors who are nonsmokers and nondrinkers, with genotype HPV16 being the most prevalent [12,13,14].

Although HPV types have a low evolutionary rate and no recent recombination events characterize their genome [15], they do present genetic variations, thus creating different viral variants [16]. Nucleotide polymorphisms are responsible for many HPV16 variants that can be divided by geographical and ethnical criteria [17], creating distinct phylogenetic clades. A recently published review summarizes the possible impact regarding the pathogenicity and immunogenicity of certain variants, concerning polymorphisms in the HPV16 *E6* gene [18].

Notwithstanding the increasing number of studies on the variability of HPV16 in cervical cancer, there are currently only a few reports describing the distribution of viral variants and their putative impact on HNC. The present study aims to investigate for the first time the E6 oncogene sequence variability of HPV16 genomes that were detected amongst the Greek population, thus providing us with further knowledge to better understand the pathogenicity, viral genome diversity and evolution of the virus.

## 2. Materials and Methods

In total, sections from 40 archival tissue samples from Greek men with mean age 57 years were retrospectively collected from patients that visited two tertiary hospitals, both located in Western Attica Athens, Greece. All patient data were de-identified and anonymized prior to any viral sequence analysis. The formalin-fixed, paraffin-embedded (FFPE) tissue samples originated from patients diagnosed by histopathology with squamous cell carcinoma of the oropharynx, larynx, tonsils, or base of tongue. All patients were nonsmokers with little or no history of alcohol abuse and were genotyped positive for HPV16 by a commercially available real time PCR assay (Sacace Biotechnologies, Como, Italy), after the DNA extraction with QIAamp DNA FFPE Tissue Kit (Qiagen, Heidelberg, Germany) following the manufacturer’s instructions. For the identification of HPV16 E6 gene variants, already published primer sets spanning the coding region between nucleotides 52 to 575 were used [19]. The HPV16 *E6* gene is positioned between nucleotides 104 and 559 and it encodes a protein of 151 amino acids [4], so the whole *Ε6* gene has been sequenced. All PCR reactions were carried out in 20 μL reaction volume using Platinum™ Taq DNA Polymerase (Thermo Fisher Scientific Inc., ThermoFisher, Antisel, Thessaloniki, Greece). Amplifications were performed in Applied Biosystems PCR System 9700 Gene Amp Thermal Cycler and the cycling conditions were as published [19]. After the removal of unused primers and nucleotides by PureLink™ PCR Purification Kit (Thermo Fisher Scientific Inc), PCR products were subjected to bi-directional Sanger sequencing using standard procedures as previously described [20] and the whole *E6* gene was available for subsequent analysis.

In order to identify nucleotide polymorphisms and amino acid substitutions into the *E6* ORF, multiple sequence alignments were performed with the MUSCLE algorithm, in MEGA X software [21]. Analyses for phylogenetic inference were conducted using Neighbor-Joining (NJ) method and trees were constructed using Kimura two-parameter correction methods of MEGA X [22]. To assess the confidence of branching patterns of NJ trees, 1000 bootstrap replicates were performed. In order to perform the sequence analysis and identify the different variants out of the 40 sequences obtained, the program Dambe [23] was used. For the phylogenetic analysis, the reference sequence of HPV16 available at the HPV16 Sequence Database (PaVE) [4] was assessed. Additionally, representative sequences of HPV16 variant lineages and sub-lineages identified to date were used for the phylogenetic analysis, including A1–3 (traditionally classified as European), A4 (Asian), B1–4 (African-1), C1–4 (African-2), D1 (North American), D2 and D3 (Asian-American), and D4 [17,24]. 

## 3. Results

The sequences obtained from the 40 isolates of HPV16 E6 gene were clustered to seven different groups GRA (4/40; 10%), GRB (3/40; 7.5%), GRC (6/40; 15%), GRD (6/40; 15%), GRE (1/40; 2.5%), GRF (1/40; 2.5%) and GRG (19/40; 47.5%), each one containing sequences with at least one unique nucleotide variation in comparison to other isolates of different groups (Table 1). The analysis of the whole *E6* gene revealed eight nucleotide substitutions, four of which (4/8; 50%) resulted in amino acid changes whilst the remaining were synonymous. The T350G variation was present in 82.5% (33/40 isolates) of the *E6* sequences. The silent nucleotide variation T185C that was found in 6 out of 40 (15%) isolates is described for the first time as a nucleotide substitution of HPV16 E6 gene in the present study. 

**Figure 1:**
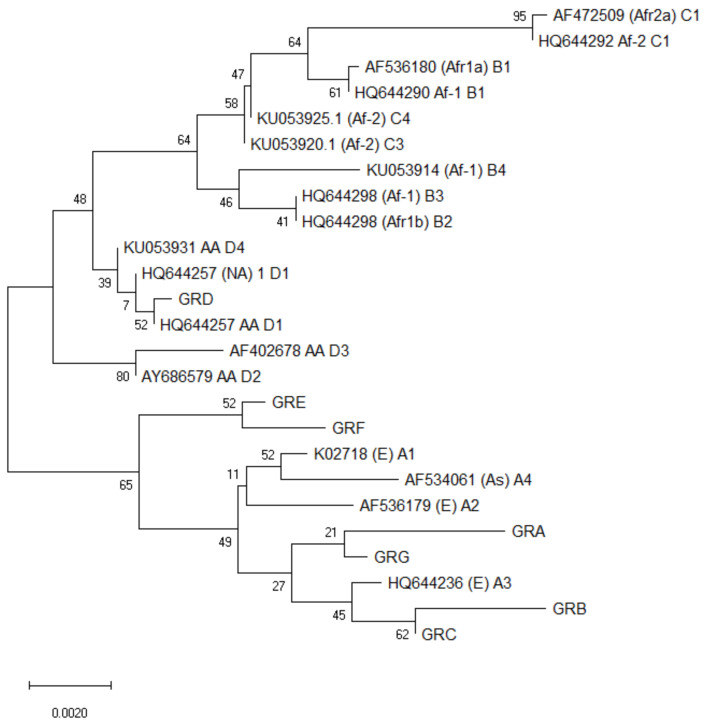
A Neighbor-Joining (NJ) phylogenetic tree (1000 bootstrap replicates) constructed using Kimura two-parameter correction methods of MEGA X, from the alignment of the HPV16 *E6* sequence from sub-lineages A1, A2, A3, A4, B1, B2, B3, B4, C1, C3, C4, D1, D2, D3, D4 and HPV16 *E6* sequences from the present study.

The phylogenetic analysis revealed that 85% (34/40 isolates) of the sequences clustered in the European variants branch. More specifically, groups GRA, GRB, GRC and GRG were closely related to the European sub-lineage A3. Although clustered in the European branch, the GRE and GRF seem to differentiate from sub-lineages of this lineage. The remaining 6/40 isolates (15%) that belong to the GRD group are clustered in the non-European branch and seem to be very closely related to sub-lineage D1 of Asian-American lineage D (Figure 1).

## 4. Discussion

Human Papillomavirus (HPV)-related oropharyngeal squamous cell carcinoma (OSCC) accounts for about 50–80% of all oropharyngeal squamous cell carcinomas [13,25]. 

When compared to tobacco-related oropharyngeal squamous cell carcinoma (HPV-OSCC), which often affects older persons (median age 64 years old) who abuse alcohol or tobacco, HPV+ OSCC’s clinical presentation is very different [26]. Younger patients exhibit distinct sexual behavior from older patients, including more oral sex partners annually and more intense sexual activity, according to Fakhry et al. [27]. However, data are contradictory and it hasn’t been proven that high-risk sexual activity and HPV+ OSCC are directly related, although oral intercourse and the quantity of sexual partners are likely to increase the risk of OSCC [28]. In comparison to HPV-OSCC which is related to smoking, HPV+ OSCC exhibits a significantly better prognosis, and the risk of mortality has been estimated to be around 60% less [29]. Regarding the molecular aspects, absence of HPV in patients with OSCCs is related to high mutational load and is characterized by multiple molecular alterations, such as deleterious mutations or loss of p53 (84%), mutation or loss of *CDKN2A* (58%), amplification of *CCND1* (31%)—which is an oncogene implicated in cell cycle regulation, amplification of *MYC* (14%) and overactivation of PI3K kinase pathway (30%) [30].

HPV16 is by far the most common HPV type associated with OPSCC and is reported in 90–95% of cases of high-risk HPV, while HPV33 is found in 3–5% and HPV18 in 2% of the HPV positive OPSCCs, respectively [12,31]. 

HPV16 variant lineages have been extensively studied, with four major variant lineages and up to sixteen sub-lineages identified to date, including: sub-lineages A1–3 (traditionally classified as European), A4 (Asian), B1–4 (African-1), C1–4 (African-2), D1 (North American), D2 and D3 (Asian-American), and D4 [17,32].

The genetic variability of HPV16 E6 oncogene is extensively studied in cervical tissue samples; specific intratypic nucleotide polymorphisms are related to the major HPV16 variant lineages which include: A1, A2, A3 sub-lineage (E6: T350G), A4 sub-lineage (*E6*: T178G), B lineage (*E6*: G132C, C143G, G145T, T286A, A289G, C335T), C lineage (*E6*: T109C, G132T, C143G, G145T, T286A, A289G, C335T, G403G) and D lineage (*E6*: G145T, T286A, A289G, C335T, T350G, A532G) [18]. 

Le Conte et al. [14] first reported sequence variability of HPV16 E6 oncogene in a total of 92 oropharyngeal samples OPSCC samples, and new mutations were pointed out. In the same study, regarding the phylogenetic relationships, individual variant types were grouped with high prevalence (90.2%) to the European and Asian lineages. A recent study, also from the United States, documented that lineage A and mainly sub-lineages A1 and A2 harbored 90.1% of the OPSCC HPV16 complete genomes [31]. 

In our study, we present for the first time preliminary data from sequencing HPV 16 *E6* gene in oropharyngeal cancer samples from a small cohort of Greek patients. One of the findings of the present study is the newly described T185C variant. The nucleotide substitutions G145T, T286A, A289G, C335T and T350G were previously found in HPV16 *E6* sequences from cervical clinical specimens originating from Greek women [33], while G176A has been previously described in Chinese cervical clinical samples [34]. The variations T350G that prevails and A131G have also been previously detected in oropharyngeal samples [14].

In accordance with the above-mentioned studies, the vast majority of the analyzed isolates were grouped in the European-Asian branch. As previously reported, HPV16 sub-lineages A1 and A2 are predominant clades of oropharyngeal cancer in the United States. Although our cohort sample is rather small, almost all samples of lineage A were closely related to sub-lineage A3. Our study population is not racially diverse; however, HPV16 E6 genomes of the non-European branch were also detected.

The nucleotide variation T350G is extensively studied in cervical tissue samples of different histological diagnoses, and the results concerning clinical aspects of this variation remain contradictory. This mutation seems to be highly tumorigenic in certain populations and is associated with severe dysplasia in some studies, whereas others report no significant correlation between the T350G variation and the development of advanced cervical dysplasia , providing conflicting outcomes even between adjacent geographic regions [33,35,36,37,38].

Having 151 amino acids and two zinc-binding domains with two C-x-x-C motifs each, the HPV-16 E6 protein is structurally important for the virus’s ability to cause cancer [39]. E6 mutation L90V is located in an interdomain region between the two zinc finger domains. As V90 is a smaller residue than L90, a decrease in the affinity of interactions with other cellular proteins is possible [40,41]. There has been considerable agreement among published studies that T350G E6 is not more effective in degrading p53. However, according to functional studies on the role of the T350G mutation, there is evidence that: this protein variant reveals a greater potential to raise hTERT expression than European Prototype E6; there is a stronger propensity for a dysplastic phenotype, as evidenced by the co-expression of both cytokeratins K5 and K10 in suprabasal cells; and differential regulation of MAPK/ERK signaling has been linked to E6 variants carrying the L90V mutation [42,43].

In the oropharyngeal samples of our small cohort of Greek patients, this variation was found to be the predominant one, which is in agreement with previously published data [14]. The high burden of this mutation in *E6* sequences of HPV16 that infects different anatomical positions demands further investigations and analyses of variants from bigger and divergent cohorts in order to indicate if there is a possible higher oncogenic capability or adaptation in a new anatomical environment.

## 5. Conclusions

In conclusion, we aimed to sequence the whole HPV16 *E6* gene from oropharyngeal specimens originated from Greek patients. To the best of our knowledge, this is done for the first time. However, our analysis involved a small number of samples only, therefore our new data regarding the phylogenies and viral genome diversity were added as preliminary results.

## Figures and Tables

**Table 1:** Nucleotide variations in HPV16 E6 ORF. A, adenine; T, thymine; G, guanine; C, cytosine; . absence of nucleotide variation or amino acid substitution.

Isolates with Identical Sequences	HPV16 Ref (K02718)	nt Position							
		131A	145G	176G	185T	286T	289A	335C	350T
	Non-Synonymous Mutations	R17G	.	D32N	.	.	.	H85Y	L90V
4	GRA	G	.	A	.	.	.	.	G
3	GRB	.	.	.	.	.	.	.	.
6	GRC	.	.	.	C	.	.	.	.
6	GRD	.	T	.	.	A	G	T	G
1	GRE	.	.	.	.	A	G	.	G
1	GRF	.	.	.	.	.	G	.	G
19	GRG	.	.	.	.	.	.	.	G

## Data Availability

Not applicable.
